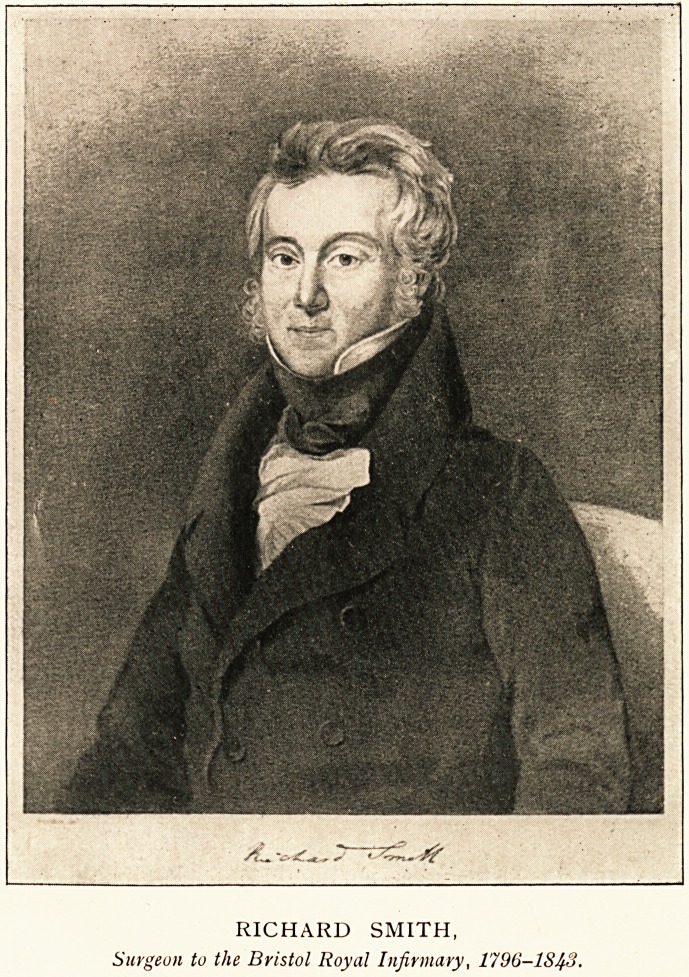# Early History of the Bristol Royal Infirmary
1Presidential Address read at the annual meeting of the Bath and Bristol Branch of the British Medical Association, May 27th, 1908.


**Published:** 1908-09

**Authors:** J. Paul Bush

**Affiliations:** Senior Surgeon to the Bristol Royal Infirmary; Lecturer on Operative Surgery, University College, Bristol


RICHARD SMITH,
*Surgeon to the Bristol Royal Infirmary, 1796–1843.*

### The Bristol Medico-Chirurgical Journal.

*"Scire est nescire, nisi id me*
*Scire alius sciret."*

SEPTEMBER, 1908.

### EARLY HISTORY OF THE BRISTOL ROYAL INFIRMARY.[1]

BY

J. PAUL BUSH, C.M.G.,

*Senior Surgeon to the Bristol Royal Infirmary;*
*Lecturer on Operative Surgery, University College, Bristol.*

SATURATED as I am with the traditions of the Bristol Royal Infirmary, after thirty-two years of work there, I think, perhaps, I might interest you to-night if I speak on "Some points in the early history of that grand old institution, the Bristol Royal Infirmary."

My friend and colleague, Mr. W. H. Harsant, in the year 1899, gave a most interesting address on "Medical Bristol in the Eighteenth Century,"[2] and at the end of this address it was freely remarked that it might have been longer, which I should consider a great compliment if accorded by my audience to this humble effort of mine.

In the year when Cheselden was living in Queen's Square, Westminster, with Alexander Pope as a patient in his house, when he was gradually bringing about the separation of the barbers from the surgeons, and vigorously working at dissection

[1] Presidential Address read at the annual meeting of the Bath and Bristol Branch of the British Medical Association, May 27th, 1908.

[2] *Bristol M.-Chir. J.*, 1899, xvii. 297.

on the bodies of malefactors; when George II. was king; at this date, in 1735, was founded the Royal Infirmary. Bristol thus claims the honour of having set to the whole of Great Britain the example of a provincial infirmary supported entirely by voluntary contributions. Previous to this nothing of the kind had been attempted out of the Metropolis, as may be seen by the following enumeration of these charities, with the dates of their foundation:—[1]

| | | | |
|---|---|---|---|
| Bristol .. .. .. | 1735 | Shrewsbury .. .. | 1747 |
| Winchester .. .. | 1736 | Liverpool .. .. | 1749 |
| Edinburgh .. .. | 1736 | Worcester .. .. | 1749 |
| York .. .. .. .. | 1740 | Newcastle .. .. | 1751 |
| Exeter .. .. .. | 1743 | Manchester .. .. | 1752 |
| Northampton .. .. | 1743 | | |

That it appears to be the oldest provincial hospital must appeal to the imagination of all interested in the rise of our profession; for there can be no doubt that the formation of these institutions was one of the greatest factors in the progress of medicine and surgery.

To many of us the mere age of the place and the numberless associations it has with the history of Bristol, to say nothing of the fact that our forbears were either subscribers, or sat on the Committee, or acted on the Staff, is sufficient to awaken the greatest veneration for it.

How many men, long since dead, have worked with heart and head and hands for the Bristol Royal Infirmary! Numbers of these are forgotten; but thanks to one character, who stands out distinctly in the history of the Infirmary, we have a unique set of records which are full of absorbing interest to the antiquary, and which throw much light on the state of medicine in the eighteenth and nineteenth centuries. This historian was Richard Smith, who was Surgeon to the Infirmary from 1796 to 1843, a lifetime.

He left fourteen portly volumes of scraps, newspaper cuttings,

[1] These dates are copied from Richard Smith's "Biographical Sketches."

portraits and gossips; from the two earliest of these volumes I have extracted small portions.

He was a man who was wrapped up in the Infirmary, and although he always did his best for the welfare of the *individual* patient at that Institution, he never lost sight of the very important duty of working for the Infirmary as a whole, a duty in these present days of increasing "individualism" that is, alas! sadly tending to diminish.

Dick Smith was a man of great virility and strength, fond of society and good wine, a great Freemason, ready to quarrel, but full of pluck and life, and ever ready to stand up for his colleagues.

The origin of the "Biographical Sketches" made by Dick Smith is due to the fact that in the year 1791 he was going round the wards with the Senior Surgeon (Mr. Godfrey Lowe) when he observed in the hands of a nurse a parcel of papers intended for the common uses of the ward. He was surprised to find that they were official letters addressed to the Governors of the Charity. He questioned her as to the means by which she obtained them, and was answered very coolly: "Where we get them all from, the old ward." Curiosity led him upstairs, and there on the floor of a deserted and ruinous garret were piles of papers, which proved to be the documents respecting the Institution from its very commencement.

The records of the General Boards and Committees were likewise thrown about, and equally liable to the depredations of the servants and patients.

Dick Smith goes on to say that "my maternal blood of the Calcotts was not a little shocked at this shameful neglect of original MSS., and although the Managing Trustees appeared to consider them of no value, I felt a regret to see them consumed by such vile uses, although as Spenser says:

> "'True it is, that when the oyle is spent
> The light goes out, and wick is thrown away.'

I determined, therefore, to make some memoranda from the books, that in case of their destruction the dates of the Society's Transactions might not be utterly lost; for this purpose I carried home to College Street the General Board and Committee Books, and at my leisure made such extracts as I wished."

Dick Smith we know was no respecter of persons, and he seems to have retained these books in his possession till September, 1794, without any inquiry being made for them, and it is perhaps lucky for us that the Institution had then a Surgeon of such determination, as it seems quite possible that if it had not been for him the greater part of our records might have been lost for ever.

Dick Smith appears also to "have *preserved*" (I will not use a stronger word) many original documents, particularly some relating to the chaplaincy, which shows us that even at this early date there was a close relationship between the healers of the soul and the healers of the body. These *borrowed* papers, together with curious old memoranda, given him by Mr. Edward Ash, Mr. Beck, Dr. T. W. Dyer, Dr. Plomer, Mr. Godfrey and Mr. Richard Lowe, enabled him to build up some of this early history.

The founder of the Infirmary was John Elbridge, who was Comptroller of His Majesty's Customs in Bristol, born at St. Anne's in the Island of Jamaica and not at the Fort, Bristol, as is generally supposed. In the year 1735 he undertook to establish the Infirmary. This he did by building two wards and furnishing them. He died February 22nd, 1738, and left £5,000 to the Institution. During his lifetime he built in his garden a school for poor girls, and left money in his will to keep this up. The "master of this school has for ever the right of sending any scholar when sick or wounded for advice, etc., by giving her a note signed Elbridge, when she is to be admitted immediately without waiting for the regular forms used by other patients, and without waiting for the proper 'take-in days.'"

A curious circumstance is that the grandson of the founder, named George Elbridge Rook, applied on March 28th, 1778, as a pauper, and was taken in by Mr. Richard Smith, senior, who amputated his leg; and in the year 1796 a descendant of the great Edward Colston became a patient at the Infirmary, and was the first patient upon whom Dick Smith performed amputation of the leg.

With regard to *practice, prescriptions,* and *prices,* it is interesting to notice that the first entry of all in the first volume is a bill :

April 7th, 1736,
Madme. Mountjoy to Jas. Bush
for two Cordials. 3/8.

which I notice was paid as soon as April 17th, 1736. I wish I could get my accounts paid as quickly as it appears some of my ancestors did in these early days.

It can here be mentioned that Parliament actually purchased of a Mrs. Stephens, at the cost of £5,000, a remedy for the solution of stone in the bladder. When this expensive recipe was afterwards published, it turned out to be "snails burned to blackness, camomile flowers, sweet fennel, parsley, and the great burdock root"; and in the year 1739 a munificent donor purchased from the said Mrs. Stephens, for the use of the Infirmary Staff, the recipe of this "precious cure" for £3 3s. od., which was rather a falling-off from the sum paid by Parliament ; but history does not relate how many calculi were dissolved by it, or how many patients were cured of their symptoms of stone at that Institution. Other "potent" drugs ordered freely in Bristol about this date (1744) were the *Toad's Powders,* which were supposed to be a cure for many ailments of men and women. They were prepared thus : "Take live toads and dip them in oil of soot, then burn in a pot with moderate heat, and pulverise."

Dr. Long Fox[1] is stated by Mr. Smith to have said: "When I came to Bristol, in about 1786, it was the practice with most of the regular physicians to wrap up the legs of patients in hot bullock's lights (lungs), and apply a pigeon split, and hot, to the soles of the feet."

Mr. Broderip, an apothecary, who practised medicine and surgery in Bristol, is stated by Dick Smith to have booked in the year :

| | | | | | £ |
|---|---|---|---|---|---|
| 1796 | .. | .. | .. | .. | 5,993 |
| 1798 | .. | .. | .. | .. | 6,931 |
| 1799 | .. | .. | .. | .. | 6,845 |

[1] The grandfather of our recently deceased colleague.—Ed.

These were the *golden* days of physic, more especially when we call to mind that there was no shilling in the pound Income Tax, and when butcher's meat was only 2d. to 3d. a pound.

The great Bristol families in these days were so wrapped up in their own particular medical men, that they could not go away for a holiday without a regular supply of physic ; and the records show that the family of Greenbys were supplied on one of these occasions by Mr. Broderip with 200 tonic draughts and 1000 pills of various descriptions. His assistant is reported to have said that " he was sick to death with a-rolling of 'em." If a draught was sent the charge would be 1/6, but if it contained musk an additional charge of 10/6 was made. One of Broderip's patients lived at Rochester, to whom he dispatched monthly by stage coach a packet of draughts containing one drachm of Tinct. Cardamom Co. and one ounce of Aqua-pump-aginis.

About 1805 the Bristol families began to find out that it was better and cheaper to go to one of the first-class physicians, and these gentlemen no doubt explained that if they alone were employed there would be no inducement to order such loads of apothecary's stuff, and that their medicines would assume a more compact and diminished form, and that they would pay less, and be spared the necessity of swallowing so many nauseous drugs. This change of fashion no doubt led to the starting of the dispensing druggists, who set up business in splendid shops in Bristol about this period, and who not only advertised the fact that the drugs they dispensed were pure, but also the fact " that a physician attended at their shops regularly at certain hours in the morning and evening and gave his advice *gratis*."

Excessive bleeding was much in favour in these early days of the Infirmary. It is reported that the celebrated physician, Dr. J. C. Prichard, of the Red Lodge, and grandfather of your President of a few years ago, who was Physician to the Infirmary in 1816, during one week ordered 96 out-patients to be " blooded," while out of 21 patients admitted under him into the Infirmary, no less than 20 required to have the strong Bristol beer let out of them in this way.

It was at the place of business of the old barber surgeons that some of the earliest of the Infirmary surgeons were educated, and there is no doubt that they had a fine experience of blood-letting, drawing teeth, and in the making-up of tinctures and pills.

*The visitors*, from the foundation of the charity, were a kind of sub-committee appointed to visit the wards and look into the general working of the Institution. I reproduce the first entry in the "Visitor's Records," dated December 19th, 1737; it is the following:—

> "Examined the beer and find it not good enough for the price."

It is not stated how the visitors examined the food and drink, but I notice that on January 1st, 1738, J. Bush—evidently the same gentleman previously mentioned—performed his duties satisfactorily, and signed the book. The writing is very shaky, but perhaps this is due to the *date* of the visit.

John Page, Surgeon to the Infirmary, 1741—1777, lived in an imposing house in Redcliffe Street, was author of various medical books, a rapid operator, and was held in great esteem by most of his fellow-practitioners. There was a report about him that he often removed fingers and toes with a carpenter's chisel and mallet, and that when he was about to operate the students used to ask one another if they were going to see "Old Johnny" play at hammer, chisel and block!"

There is no doubt that as a lithotomist he was skilful and successful, having cut more patients for stone, both in private and in the Infirmary, than any surgeon of his time in Bristol. He was the first in Bristol to practise the lateral operation. Surgeons in these days had to bring their own forceps, and his were not always of the latest pattern. As he completed one day one of these brilliant operations, the *junior* surgeon present (whose duty it was to hold the patient's legs and not to talk) said to him, "You will be retiring soon, sir, will you not? When you do, you must make us a present of your forceps, for I believe they know the way into the bladder of their own accord." This remark might be taken in two ways. Dick Smith gives the junior the benefit of the doubt, and puts it down to the spontaneous effect of the pleasing sensation which all the bystanders, in common with the patient and the operator, feel at the sight of the stone.

JAMES FORD, Surgeon to the Infirmary, 1743—1759, son of Canon Ford of Wells, a Bristol student of medicine, was a man of considerable talent and fascinating manners. Ford was making his mark as a surgeon in Bristol when he attended Lord Bute, the Premier, who was taking the waters at Hotwells. Lord Bute persuaded him to leave Bristol and go to London, where in 1788 he was honoured by Queen Charlotte, the wife of George III., nominating him to be her physician. The Royal Warrant is bound up with Dick Smith's "Records." He became one of the leading men in London and made a considerable fortune.

He was a friend of David Garrick and Sheridan, and in conjunction with Lindley and Sheridan purchased for £35,000 Garrick's share in Drury Lane Theatre. Lindley was unwilling or unable to make good his payments, and Sheridan brought no money into the transaction, so that poor Ford lost his fortune.

I have found the following anecdote. Soon after Dr. Ford had made the purchase from Garrick, the King saw and asked him if it were a fact? Dr. Ford replied, "Yes, may it please Your Majesty." "Oh, yes," replied the King, "it pleases me well enough, quite right, quite right, all very proper and appropriate, for Charlotte *assures* me that you are quite at home behind the *curtain*."

James Ford on retiring from Court practice was able to get his brother John appointed, and so Bristol was for a second time honoured by the King.

John was Surgeon to the Infirmary from 1759—1775, when he was made an M.D. by the Archbishop of Canterbury.

The following correspondence is of interest :—

ALBERMARLE STREET,
*February 23rd*, 1771.

GENTLEMEN,

It being my intention to give my son an introduction to my own business, it will be necessary for him to finish his education in London and abroad, and as that will take up a considerable time, it will be much to his advantage if he can be permitted to leave the Infirmary at the expiration of his *sixth* year. I therefore humbly request this indulgence of you, which will greatly add to the favours you have already conferred on him.

Gentlemen,

Your most obliged and most obedient servant,

J. FORD.

This letter shows how highly esteemed the Infirmary was in 1771, as here we have such a noted man as James Ford, physician to George III., sending his son to Bristol to receive the first six years of his medical studies, these to be followed by special courses in London and Paris. In another letter the father states, "I feel sure that at the Bristol Infirmary he will learn nothing that hereafter I shall wish him to forget."

And again, "From the general knowledge I have of the great hospitals, and the thorough one I have of yours, I know he cannot be better placed than there."

It also shows us that the usual period of training for a medical student in those days was some seven or eight years.

This letter shows what the son thought of the Infirmary:—

GENTLEMEN,

I cannot quit this place without returning you my thanks for the many civilities you have shown me during the course of my apprenticeship, and the advantages I have reaped there under your countenance and protection, and I beg leave to assure you that I shall always rejoice at promoting the interest of this excellent Infirmary, endeared to me as it will always be by a remembrance of the many happy hours I have spent in it, and the *genteel behaviour* I have ever experienced from each individual belonging to it.

I have the honour to be, gentlemen,

Your much obliged and very humble servant,

JAMES FORD.

INFIRMARY,

*September* 18*th*, 1772.

JEROME NORMAN, Surgeon to the Infirmary, 1755—1762. It was between these dates that at a consultation at the Infirmary Norman proposed to amputate a leg at the hip-joint for hip-joint disease. All the other surgeons were so horror-struck at the very suggestion of such an operation, that they refused to give their consent, and so the patient was allowed to die without a chance being given him. We have, therefore, to lament the fact that Bristol did not have the privilege of being the first to perform this major operation, as it was not till the year 1776 that Mr. Thompson actually performed it in England.

JOHN CASTELMAN, Surgeon to the Infirmary, 1755—1779, was a well-bred gentleman. His father having lost some £40,000 in the South Sea Bubble, the son had to make his own living. He lived in Prince's Street. He had a hard fight to get on the Staff, and the election was one of the fiercest on record. The Press teemed with letters and communications, some extolling the various virtues of one or other of the six candidates, but many full of the most low and personal scurrility. It was freely advertised that one candidate was filling the streets of Bristol with "wooden legs," and that another had cured many cases which had been dismissed from the Infirmary as incurable.

Castelman was, however, elected by the small majority of twelve votes. It was this gentleman that made the somewhat offensive remark with regard to the operative powers of Mr. John Page already referred to.

It does not appear that his surgical abilities were of a high order. He is reported to have been a man who was content to let the art of surgery remain where he found it. He was a man of no nerve, and in operations which were not absolutely commonplace he was frequently foundered, and his colleagues had more than once to finish an operation for him.

WILLIAM BARRETT served his apprenticeship time to old Rosewell, the barber-surgeon, where also many noted medical men of Bristol commenced their training. He was an unsuccessful candidate when Castelman, Townsend and Norman were elected in 1755. He practised surgery and midwifery in the city with the highest reputation for professional skill. His health gave way under the strain of his profession, and he retired to Wraxall about 1787 to write the *History of Bristol*, a well-known work. It was to Barrett that Chatterton applied for a certificate of his abilities to go to Africa as a surgeon, but as Barrett refused this certificate, the unfortunate young man (Chatterton) did not forget him when he wrote a poem in 1770 upon the profession in Bristol.

Old SAM PYE, whose "home-bred documents" are sneered at by Chatterton in the poem referred to, belonged to a family which without any interruption may be traced as practising surgery in Bristol for at least a century before 1759.

The description of him is this: "He was a man who, by his experience, successful operations, and peculiar sagacity of genius in treating the more obscure diseases, advanced surgery and midwifery to its greatest height." He died in 1759, aged 74 years.

There were numerous *Sam* Pyes, fathers and sons. One of them wrote a book on lithotomy; many of them were candidates for the post of surgeon at the Infirmary, but none were elected.

Dr. EDWARD LYNE, Physician to the Infirmary, 1758—1772, was the first to advocate "Bristol milk." He described it as "a generous wine from Lisbon," and of which he partook one bottle always for dinner each day.

As far as I know, he was the first Bristol man to become a pure consultant, and I believe he always refused to see any patient after 2 p.m.

He was an alderman of the city, and his mode of thinking of his personal comfort and convenience is shown by his letter of resignation. He says:—

GENTLEMEN,

The Infirmary interferes so much with my private affairs that I cannot possibly attend to all. I therefore resign, and am

Your obedient servant,

EDWARD LYNE.

THE INFIRMARY IN 1762.—In-patients admitted 1,157, and 386 were refused admission for want of room. Average number of patients in the house 132, not including a large number which were placed in lodgings at the expense of the Infirmary. Of these 717 were cured, and 103 died during this year.

In 1781 the foundations of the present building were laid. In 1788 the east wing was completed, and in 1805 £10,000 was collected for the west wing. In 1850 the Infirmary obtained the permission to call itself the Bristol Royal Infirmary.

It is clear from these records we had to wait from 1735 to 1805 before there was sufficient money to build a suitable Infirmary for such an important city as Bristol. There was during these years heaps of money in the old port. I notice that the merchants on one day in 1745 subscribed their names to sums amounting to £40,000, to repell a large body of Highlanders led by the Pretender's son, and in July of the same year the contents of two Spanish prizes taken I expect by Bristol ships, and valued at just under a million pounds sterling, were lying in Bristol and afterwards sent to London. Some of this might, in my opinion, have been devoted to the Bristol Infirmary.

By a note February 21st, 1739, it appears that the Infirmary was in the habit of paying various sums, such as 4/6 or 2/6, for carrying patients to their place of abode, thus showing that even at this early date patients came from a considerable distance. On March 17th in the same year 5/- was paid to carry James Bruan to Salisbury, his place of abode.

Mr. LUDLOW was a very noted man, for at the election of 1766 for *one* surgeon Mr. Ludlow received 146 votes and Mr. Thomas Stone 147 votes. A recount was demanded by Mr. Ludlow, and after much uproar the chairman stated that Mr. Ludlow had 147 and Mr. Stone had 146 votes. A third count was demanded. While this was proceeding the two sides came to blows, the voting papers were upset and some lost. What was to be done ? The rules said they must that day elect, and the rules also said only one must be elected. The chairman (Sir Abraham Elton), however, ruled that they were both elected.

The records state that at this election a certain doctor gave such drastic medicine to three of the supporters of Mr. Ludlow that they were unable to vote ; while a supporter of Mr. Stone during the count was perceived to make an attempt to secrete a voting paper in his hat.

Mr. Ludlow got into great trouble with the physicians for treating medical cases without an M.D. degree, in fact he was prosecuted in the courts by his colleague, Dr. Rigge, for so doing. The case ended, I find, by his obtaining an M.D., and the following words occur :—

"The Jury being satisfied of his great abilities as a medical practitioner, did not enquire whether he had sent for it by post from Scotland, or whether like his opponent he had walked for it to Padua."

Dr. RIGGE, Physician, 1767—1777. Dr. Rigge had only been elected a few years when he requested Mr. John Page, who had been a surgeon there for thirty years, to bleed a patient himself. John Page sent his dresser, and so the row began.

He created a great ferment in the Infirmary by trying to persuade the General Board that the surgeons should not admit cases without the consent of the physicians. Dr. Rigge also maintained that the surgeons should not perform any major operations without the consent of the physicians.

A row over the apothecaries caused Mr. Richard Smith to challenge Dr. Rigge to fight on Brandon Hill. They met, and were about to fire, when the seconds advanced and made one more appeal to prevent bloodshed, as each medical man had a wife and family. The duel was prevented, Dr. Rigge made an apology, but both parties held a sullen reserve towards each other ever after.

The following letter ends the career of Dr. Rigge at the Infirmary. It reads thus :—

BRISTOL,

*February* 18*th*, 1778.

GENTLEMEN,

During Mr. Bridges's lifetime it was a pleasure to attend the Infirmary, but since we unfortunately lost him, there has been great disorder, and confusion, collusive innovations, &c., and as the discord and difference of opinion, still subsisting among the gentlemen of the House, afford but little prospect of putting it upon a more respectable footing, I therefore resign my office, and am,

Gentlemen, your most humble and most obliged servant,

THO: RIGGE.

Complaints against members of the Staff were recorded. This is of interest as showing that 130 years ago patients, who paid nothing for their treatment, were then just as ready as now to bring false accusations against the Staff.

The diet table of 1785 is very interesting. With regard to common diet, it was only on Monday nights the patients were provided with *nightmare* in the form of cheese for supper.

In the low diet *panado* is bread soaked in water, *pippin* I suppose is apple and water, *cyder whey* is always excellent, and *baum* is evidently *balm*, of which John Evelyn says: "Balm is sovereign for the brain, strengthening the memory, and powerfully chasing away melancholy."

I notice how largely *beer* entered into the diet of these days, as in common diet, although meat was only allowed on two days of the week, four glasses of beer *per diem* were given to each patient.

An interesting caricature was freely circulated during the election of 1781. It shows "knock-kneed Billy" introducing Mr. Richard Smith, senior (the grandfather of Dick Smith) to the Devil. He says: "Here is our staunch friend Dick, the surgeon, who wishes to be introduced to your Infernal Majesty." The Devil replies: "My dear knock-kneed Billy, I have long known the merit of this cutter and slasher." Someone questioned at the time whether the caricature of Dick Smith was meant for him, as he did not appear to be opening his mouth (Smith being a great talker).

I cannot close without expressing my indebtedness to Dr. Newman Neild for his kindly assistance in the preparation of the numerous lantern photographs of early physicians and surgeons in the costumes of the day, as well as reproductions by the same method of diet cards, admission cards, Royal warrants, views of the Infirmary buildings, and election caricatures.[1]

[1] The address was rendered most interesting by the exhibition of many pictures, of which we reproduce one, the portrait of Richard Smith.

## Figures and Tables

**Figure f1:**